# Alarmins in Osteoporosis, RAGE, IL-1, and IL-33 Pathways: A Literature Review

**DOI:** 10.3390/medicina56030138

**Published:** 2020-03-19

**Authors:** Massimo De Martinis, Lia Ginaldi, Maria Maddalena Sirufo, Giovanni Pioggia, Gioacchino Calapai, Sebastiano Gangemi, Carmen Mannucci

**Affiliations:** 1 Department of Life, Health, & Environmental Sciences, University of L’Aquila, 6700 L’Aquila, Italy; demartinis@cc.univaq.it (M.D.M.); lia.ginaldi@cc.univaq.it (L.G.); maddalena.sirufo@gmail.com (M.M.S.); 2 National Research Council of Italy (CNR)-Institute for Biomedical Research and Innovation (IRIB), 98164 Messina, Italy; giovanni.pioggia@cnr.it; 3 Department of Biomedical and Dental Sciences and Morphofunctional Imaging, University of Messina, 98125 Messina, Italy; gcalapai@unime.it; 4 School and Division of Allergy and Clinical Immunology, Department of Experimental Medicine, University of Messina, 98125 Messina, Italy; gangemis@unime.it

**Keywords:** osteoporosis, alarmins osteoporosis, RAGE osteoporosis, HMGB1 osteoporis, S100 calgranulin proteins osteoporosis, IL-1 osteoporosis, IL-33 osteoporosis

## Abstract

Alarmins are endogenous mediators released by cells following insults or cell death to alert the host’s innate immune system of a situation of danger or harm. Many of these, such as high-mobility group box-1 and 2 (HMGB1, HMGB2) and S100 (calgranulin proteins), act through RAGE (receptor for advanced glycation end products), whereas the IL-1 and IL-33 cytokines bind the IL-1 receptors type I and II, and the cellular receptor ST2, respectively. The alarmin family and their signal pathways share many similarities of cellular and tissue localization, functions, and involvement in various physiological processes and inflammatory diseases including osteoporosis. The aim of the review was to evaluate the role of alarmins in osteoporosis. A bibliographic search of the published scientific literature regarding the role of alarmins in osteoporosis was organized independently by two researchers in the following scientific databases: Pubmed, Scopus, and Web of Science. The keywords used were combined as follows: “alarmins and osteoporosis”, “RAGE and osteoporosis”, “HMGB1 and osteoporosis”, “IL-1 and osteoporosis”, “IL 33 and osteopororsis”, “S100s protein and osteoporosis”. The information was summarized and organized in the present review. We highlight the emerging roles of alarmins in various bone remodeling processes involved in the onset and development of osteoporosis, as well as their potential role as biomarkers of osteoporosis severity and progression. Findings of the research suggest a potential use of alarmins as pharmacological targets in future therapeutic strategies aimed at preventing bone loss and fragility fractures induced by aging and inflammatory diseases.

## 1. Introduction

Osteoporosis is a progressive disease characterized by a decrease in bone mass and microarchitectural deterioration of the bone structure. This condition can compromise skeleton physical strength, thus increasing susceptibility to fractures or minor trauma. Bone tissue is continuously remodeled throughout life by the integrated action of the bone cells. An imbalance between bone formation and bone resorption is responsible for the onset of osteoporosis. Osteoporosis is a multifactorial disease with various combined etiopathogenetic mechanisms [1]. Estrogen deficiency in postmenopausal women and the aging process, as well as several age-related inflammatory diseases, induce bone loss and osteoporosis, mainly increasing the production of pro-inflammatory and osteoclastogenic cytokines and immunoregulatory molecules which drive bone resorption [2]. Clinical and molecular evidence indicates that estrogen-regulated cytokines affect bone turnover. They are considered the primary mediators of the accelerated bone loss that occurs at menopause [3]. Similarly, cell senescence and immune system reshaping associated with aging influence bone remodeling, leading to osteoporosis [4]. Based on these recent discoveries, current and emerging drug therapies for osteoporosis also target cytokines, regulatory molecules, and their receptors [5].

Alarmins are signaling mediators of inflammatory responses after infection, trauma, and injury [6]. They function as intercellular defense signals through interaction with chemotactic factors and pattern recognition receptors (PRRs) to stimulate immune cells in host defense through the binding of specific parts of the pathogen, called pathogen-associated molecular pattern molecules (PAMPs) [7]. Mannose receptor (MR), toll-like receptors (TLRs), and NOD-like receptors (NLRs) are examples of PRRs. Engagement of PRRs with PAMPS stimulates the release of pro-inflammatory cytokines, triggering inflammation. Through PRRs, the innate immune system has the ability to sense tissue damage by recognition of mislocalized or altered endogenous molecules known as damage-associated molecular patterns (DAMPs), a term that can be used interchangeably with alarmins [6]. 

Alarmins, mainly derived from innate immune cells, merge intracellular functions correlated to cell homeostasis and extracellular cytokine capabilities, thus leading to inflammatory responses by different mechanisms such as recruitment of immune cells, stimulation of adaptive immunity, and starting of multiple feedback loops to increase or modulate inflammation and ultimately initiate tissue repair [7]. Alarmins share conserved regulatory mechanisms, such as secretory routes, enzymatic processing, and post-translational modifications, that regulate their extracellular functions. Their release from mesenchymal cells plays a key role in allowing the immune cells to be alerted to tissue damage [8]. 

Although they share many biological characteristics, alarmins differ in many respects, primarily with regard to their respective receptors. Until now, a number of alarmins have been identified [9], including, among others, high mobility group box 1 and 2 proteins (HMGB-1 and 2), S100 proteins, IL-1, and interleukin (IL)-33, that from recent studies seem to be variously involved in skeletal biology [8,10]. These alarmins recognize different kinds of receptors which variously mediate their functions. In particular, the receptor for advanced glycation end products (RAGE), whose role in bone homeostasis and osteoporosis is starting to be discovered [11], is a PRR binding several endogenous and exogenous ligands, including HMGB-1 and 2 and S100 proteins. IL-1 binds the inflammatory receptor type I and the suppressor receptor type II. In contrast, IL-33 binds a different receptor, the ST2 receptor, a member of the interleukin 1 receptor family. The ST2 has two isoforms: a membrane-bound receptor form (ST2L) and a soluble form (sST2), which functions as a soluble decoy receptor [12,13].

In this review, we performed a literature review of the current evidence on the RAGE signaling pathway and the roles of HMGB, S100, IL-1, and IL-33 alarmins in bone turnover and osteoporosis. 

## 2. RAGE

RAGE, a multiligand receptor, belongs to the immunoglobulin receptor superfamily that plays a critical role in innate immune response regulation [14]. It is a 50 kDa protein consisting of an extracellular N-terminal V-type (variable) and two C-type (constant) domains, a single transmembrane domain, and a C-terminal cytoplasmic tail. The receptor ligand-binding portion is the extracellular V-type domain, while the cytoplasmic tail is necessary for signal transduction as well as adaptor protein association. RAGE has been identified in two extracellular secreted forms: an endogenous secretory (es) form and a soluble (s) form [15]. Even if the sRAGE isoforms are missing transmembrane and cytoplasmic domains, they maintain their ligand-binding ability, acting as decoy receptors and inhibiting the binding between RAGE with their ligands or with other receptors [16]; consequently, sRAGE treatment causes a downregulation of RAGE signaling, thus decreasing inflammation [17]. 

Nevertheless, sRAGE administration can cause a stimulation of signal transduction, thus inducing an inflammatory response [18]. The diagnostic role of sRAGE as a biomarker has been debated due to conflicting results. A correlation between circulating sRAGE levels and osteoporosis has been described [19], but low serum esRAGE levels may also be indicative of high bone resorption and vertebral fracture risk [20] because of the lack of inhibitory sRAGE decoy effects. It has been proposed that increased levels of circulating AGE and esRAGE are related to an increase in bone turnover and hip fracture frequency in elderly men, correlating with osteopenia and osteoporosis [21]. sRAGE levels are also negatively correlated to BMI and leptin level, suggesting sRAGE as a good biomarker of lipid metabolism and bone fragility.

RAGE is expressed in several cell types, such as mesenchymal stem cells, osteocytes, osteoblasts, and osteoclasts, in which its expression is differently regulated during cell proliferation, differentiation, and survival processes [22]. 

RAGE binds endogenous DAMPs, including AGEs, S100 calgranulin proteins, HMGB1, amyloid β peptide, macrophage 1 antigen, and heat shock proteins, released in response to cell stress and death extracellularly [23]. RAGE stimulates, after ligand binding, downstream signaling pathways involving AP-1, NFAT, NF-κB, STAT3, and CREB transcription factors, inducing cytokine/chemokine transcription and regulating the apoptosis and autophagy process [24]. Interestingly, such transcription factors are also implicated in the RANK/RANKL pathway driving osteoclastogenesis [25]. Moreover, cell-/tissue-specific gene transcription and cell-type-related differences in adaptor protein signaling can contribute to cell-type-specific RAGE activation [26]. Even metabolic status, reactive oxygen species levels, and intra- and extracellular redox conditions may alter RAGE ligand properties, thus modifying downstream signaling [27,28].

RAGE plays a key role in the regulation of bone metabolism under physiological conditions and can contribute to the onset of several bone-related diseases, including osteoporosis [11,23]. However, the specific mechanism of RAGE involvement in bone homeostasis and pathology is not clear. It has been proposed that RAGE activation alters numerous downstream signaling pathways, in particular, αvβ3 integrin-mediated adhesion in osteoclasts [29]. Evidence suggests that RAGE-deficient osteoclasts show a reduction in integrin expression, necessary for osteoclast maturation and adhesion, so osteoclasts cannot respond to vitronectin and show altered PYK2 and ERK1/2 phosphorylation. A decrease in c-Fos and NFATc-1 expression in response to M-CSF and RANKL signaling has been also proposed. AGE–RAGE activation increases Raf/MEK/ERK signaling in osteoblasts, causing a decrease in cell viability and activation of beclin-1 and LC3B-mediated autophagy [30]. Therefore, RAGE is an important factor for signaling events stimulating both osteoclast and osteoblast differentiation and function. RAGE insufficiency causes a suppression of PPARα and its co-factor PGC1α, leading to a pro-inflammatory condition and to a bone mineralization decrease [31].

An increase of RAGE ligand signaling is related to different pathologies characterized by reduced bone mass/strength, including diabetes, cancer, cardiovascular disease, and neurodegeneration [32]. Although RAGE expression is upregulated in inflammatory pathological conditions, it has been suggested that RAGE signaling may have also a role in inflammation resolution and tissue repair [33]. The harmful effects of the RAGE ligands AGEs on osteoblast viability and activity have been identified [11]. Similar findings have been reported for HMGB1. It is suggested that high HMGB1 levels are associated with osteoblast and osteocyte apoptosis, while low-level/short-term administration may stimulate osteoblast differentiation and bone formation, thus promoting fracture healing. On the contrary, an inhibitory effect of direct RAGE ligand exposure on osteoclasts has been proposed [11]. 

AGEs originate from aldose sugar-mediated nonenzymatic protein and lipid chemical modifications [34]. It has been proposed that during aging and hyperglycemia conditions, an increase in AGE formation and tissue accumulation occurs. Particular susceptibility to this nonenzymatic modification in tissue characterized by long-lived protein with low turnover has been proposed [35]. Under diabetic conditions, lipid, nucleic acid, and protein modification caused by AGEs changes cellular integrity and function, evoking oxidative stress and inflammation through RAGE interaction. Protracted AGE signaling may have an important role in the onset and progression of osteopenia related to diabetes and to aging-related bone fragility [36,37]. 

In view of this evidence, the possible therapeutic effects of RAGE targeting in various inflammatory diseases, including osteoporosis, has been investigated.

In the last decade, several RAGE inhibitors have been proposed, but their therapeutic potential in osteoporosis is still uncertain [11]. The effects of targeting/blocking RAGE and its ligands in bone have been evaluated in numerous studies [38]. Several compounds have been proposed, such as the small molecule TTP488 (azeliragon) that inhibits RAGE’s ability to bind ligands [39]. Another RAGE-inhibiting compound, FPS-ZM1 [40], has shown a protective effect in bone, preventing mitochondrial dysfunction and apoptosis mediated by RAGE in osteoblasts and osteocytes. FPS-ZM1 rescues AGE-induced loss of the osteogenic potential of adipose-derived stem cells (ASCs) in a diabetic environment [41]. Irbesartan, an agiotensin II type 1 receptor blocker, has anabolic bone effects in diabetes, inhibiting the deleterious effects of oxidative stress mediated by AGEs/RAGE [42].

Attention has also been paid to aptamers—short single-stranded RNA or DNA oligonucleotides able to bind several types of proteins with high affinity and specificity. Aptamers can be easily generated and they are able to highly penetrate into organs with a low risk of allergic reactions and with a greater specificity than antibodies for neutralizing and/or blocking target proteins or cell surface receptors [43]. The pathogenetic mechanism of postmenopausal osteoporosis also involves oxidative stress and collagen modifications, which lead to unstable bone remodeling processes through RAGE signaling, causing stimulation of bone resorption and inhibition of bone formation. Since oxidative stress promotes AGE formation, inhibiting normal enzymatically derived crosslinking and degrading the collagen structure with an increase in fracture risk, antioxidant therapies have been proposed to interfere with this positive feedback loop mediated by RAGE signaling in postmenopausal osteoporosis [44].

## 3. Alarmins

Alarmins are endogenous molecules with multiple functions, which are detected by the host immune system and contribute to host defense. Alarmins are constitutively expressed and can be considered the first defense against infections and traumatic insults. They initiate an innate adaptive immune response thanks to their capacity for recruiting leukocytes and activating dendritic cells (DCs). Alarmins stimulate DC maturation, allowing their migration and delivery in draining lymph nodes to the T lymphocytes, their antigenic contents thus resulting in adaptive immunity and T-cell-dependent long-term immune memory [45]. Alarmins have been found bound to cell granules, cell chromatin, cytosol, and vesicles, but it has been suggested that any nucleated cell may release them; moreover, their targets are chemotactic GiPCR and several activating receptors expressed by different cell types. 

Several alarmins have been identified; here, we focused our attention on alarmins involved in various bone remodeling processes and in the onset and development of osteoporosis, and their potential role as therapeutic biomarkers.

### 3.1. HMGB1

The high-mobility group (HMG) proteins belong to the low-molecular-weight family of nonhistone chromatin-associated proteins. They are able to regulate the DNA transcription, replication, recombination, and repair processes [46,47,48]. The HMG can be divided into three groups of proteins: HMGA, HMGB, and HMGN. HMGB proteins are secreted by macrophages [49,50] and by RAW-C3 cells. Their secretion is enhanced in RAW-C3 cells treated with IL-1/TNFα and RANKL.

The HMGB proteins are mainly present in the nucleus, but it has been shown that a small fraction of the nuclear HMGB proteins are probably bound to the RRS on the TNFα promoter. 

HMGB1 and HMGB2 are highly homologous proteins belonging to the HMGB subgroup.

HMGB1 is prevalently localized in the nucleoli, while HMGB2 is primarily present in the cytoplasm and nucleus. Moreover, it has been demonstrated that HMGB1 is secreted by an atypical endolysosomal-like pathway, after RANK-L stimulation, in response to bacterial infection and inflammation, and it is highly expressed in cells during embryonic and postnatal life [47,49,50]. HGMB2 is associated to mesenchymal differentiation, and an involvement in early articular cartilage degeneration has been demonstrated [51].

HMGB2 expression is restricted to the spleen, thymus, and testis in adults [52,53,54]. HMGB1 and HMGB2 are both necessary for osteoclast formation and TNFα expression. 

HMGB1 plays the role of alarmin in several tissues, but it is in fact released by dead and dying cells to alert the innate immune system to damage and tissue repair. Moreover, extracellular HMGB1 is a ligand for TLRs and RAGE, which amplify the inflammation process. HMGB1 is also considered a bone-active cytokine: when released, HMGB1 enhances the expression of RANKL, IL6, and TNFα in osteoblastogenic bone marrow stromal cells and constitutes a chemotactic stimulus to osteoclasts. HMGB1 also has a role at the immune–bone interface as an osteocyte alarmin and mediator of normal remodeling and inflammatory bone loss. Apoptotic osteocytes—the terminally differentiated osteoblasts embedded in bone—release signals that enhance bone resorption by osteoclasts and target their migration. This phenomenon, which underlies the normal repair of microdamaged bone, also contributes to bone loss in immunosenescence [55] and inflammatory conditions [56]. 

Experimental evidence shows that HMGB1 is expressed in primary osteoblasts and osteoclasts. In particular, it has been proposed that HMGB1 signaling in MSCs/osteoblasts promotes tooth socket bone healing in mice following a tooth extraction [56], HMGB1 stimulates cytokine release and promotes osteogenic MSC differentiation [57]. 

Moreover, HMGB1 drives osteoblast migration and promotes fracture site vascularization, stimulating endochondral bone formation [58,59]. 

The impact of HMGB1 in the bone microenvironment has been proposed. Some evidence showed the effects of recombinant protein rHMGB1 on multiple murine bone cell preparations. Their results suggest that rHMGB1 enhanced the RANKL/OPG steady state mRNA ratio with a consequent improving of release of tumor necrosis factor-alpha and interleukin-6 in osteoblastogenic bone marrow stromal cell cultures but not in the calvarial-derived MC3T3-E1 cells. Moreover, a promotion of GSK-3beta phosphorylation in MC3T3-E1 cells mediated by rHMGB1 has been suggested but not in BMSCs. The authors conclusion suggested that apoptotic bone cells release HMGB1, that within the marrow HMGB1 is a bone resorption signal, and that intramembraneous and endochondral osteoblasts exhibit differential responses to this cytokine [60].

The effect of the HMGB1-mediated ERK pathway on the healing process of bone fracture through constructing the rat tibial fracture models, has been proposed. Experimental results suggest that HMGB1 could promote the expressions of osteogenesis-related genes and accelerate the healing process of fracture via ERK signaling pathway activation [61].

Moreover, other experiments showed that RANKL treatment stimulates the HMGB1 and HMGB2 proteins to bind the RANKL-responsive sequence and upregulates TNFα transcription. A direct correlation between the expression of HMGB and TNFα and osteoclast formation in TSHR-null mice and TNFα-null mice has also been proposed, thus concluding that HMGB and TNFα play critical roles in the regulation of osteoclastogenesis and the remodeling of bone [62].

Evidence supporting the role of extracellular HMGB1, as an autocrine ligand of RAGE, in RANKL-induced osteoclastogenesis in vitro and in vivo have been proposed [63]. It has been also suggested that extracellular HMGB1 stimulates the differentiation of osteoclasts precursors in the presence of low levels of RANKL in in vitro and in vivo experiments [63].

### 3.2. S100 Calgranulin Proteins

The S100 proteins are small molecules (10–12 kD) belonging to a highly conserved family of proteins consisting of 24 members distinguished by their different functions. It has been proposed that some of them exert intracellular regulatory effects, others possess both intracellular and extracellular functions, and others mainly have extracellular regulatory effects [64]. The involvement of S100 proteins in the regulation of differentiation, energy metabolism, proliferation, inflammation, apoptosis, migration/invasion, and Ca^2+^ homeostasis processes has been proposed. This action has been correlated to interactions with several targets such as receptors, cytoskeletal subunits, enzymes, transcription factors, and nucleic acids. The ability of S100 proteins to control cell differentiation, proliferation, migration, and survival in normal or pathological conditions, or in tissue repair has been proposed [64]. S100 proteins are linked to several human pathologies, such as cancer [58,65], neurodegenerative diseases [66,67], autoimmune diseases, and arthritis [68]. Moreover, a role of S100 protein in the cartilage repair process and osteoarthritis has been proposed [64,69,70].

S100 proteins are released by leukocytes during inflammation, and they act through RAGE (S100A12 and S100B) and/or TLR4 (S100A8, S100A9), requiring Ca2+ [71,72]. 

S100 family members can activate or inhibit receptors in a tissue- and context-specific manner. It has been proposed that RAGE–S100 protein signaling can promote the release of cytokines in endothelial cells and leukocytes through stimulation of NF-kB, thus causing a proinflammatory condition and contributing to inflammation-related diseases [73]. 

S100 proteins regulate skeletal metabolism in bone via direct and indirect action on bone cells [65,74].

It has been proposed that extracellular S100A4 can inhibit by NF-κB activation, osteoblasts mineralization function causing an imbalance in bone homeostasis by inhibiting new bone formation [66,75]. 

Further, S100A8 activates osteoclasts by interacting with the toll-like receptor 4 mediating osteoclastic bone destruction in experimental arthritis [76], and it has been proposed that S100A9 treatment in osteoblasts stimulates RAGE expression and promotes cytokine release. Additionally, S100A9-treated osteoblast CM increased osteoclast differentiation/activity, whereas directly in osteoclasts S100A9 inhibited osteoclastogenesis [77].

The protein S100A16A is a novel member of the S100 family; it is ubiquitously expressed in different tissue types and it is linked to several human diseases, such as prostate cancer, obesity, and inflammation [71]. 

Recent evidence suggests a role of S100A16 in osteoblast differentiation. S100A16 inhibits osteogenesis and stimulates adipogenesis with a consequent increase in PPARc expression and a decrease in RUNX2 expression during transcription [78]. 

### 3.3. IL-1

IL-1 is considered a key immunoregulatory and proinflammatory cytokine, produced by the inflammasome, a caspase-1 activating molecular platform, in response to selected danger-associated molecular patterns and pathogen-associated molecular patterns [79,80].

IL-1, known as “osteoclast activating factor,” is a multifunctional cytokine able to regulate different cellular tissue functions including osteoclastogenesis. It signals on osteoclast lineage cells to increase osteoclast viability and resorptive capacity, and it is able to enhance RANKL-induced multinucleation of osteoclast precursors. However, IL-1 can also act independently of RANKL [81].

IL-1 increases bone resorption by upregulating both differentiation and activation of osteoclasts as well as by stimulating the release of degradative products, such as matrix metalloproteinases [82]. 

There are at least two types of IL-1, L-1 α, and IL-1 β, performing similar activities, but encoded by different genes, and two types of IL-1 receptors, the stimulatory receptor (IL-1R1, type 1) and the suppressive receptor (IL-1R2, type 2), both expressed on osteoclasts in the normal healthy bone. In contrast, IL-1 stimulatory receptor type 1 is preferentially expressed in pathologically activated osteoclasts, performing severe bone destruction with markedly suppressed expression level of IL-1 inhibitory receptor type 2. The IL-1 receptor antagonist (IL-1 RA) binds to both the receptors, blocking their effects. Deletion of the gene encoding IL-1 receptor antagonist, that functions as a natural IL-1 inhibitor, produces spontaneous polyarthropathy with bone erosions [83]. Moreover, IL-1 interacts synergistically with other cytokines, including TNF, IL-6, IL-17, and IL-31 in inducing upregulation of bone resorption and osteoporosis [84]. 

Osteoclasts that show pathological bone destruction are different from normal osteoclasts, in particular, IL-1 induced pathological osteoclasts likely possess several resorption areas which contribute to an extremely high resorption activity in the presence of IL-1 [85]. 

Canonical osteoclast differentiation is initiated by RANK receptor signaling to activate the transcription factors NFATc1 and cFos, which in turn increase IL-1R expression. Thus IL-1 activates osteoclast-specific genes in osteoclast precursors to potentiate osteoclast formation. Moreover, IL-1 has been reported to enhance mainly “pathologically activated osteoclasts” that favor bone loss [86].

IL-1 promotes osteoclastogenesis through synergistic signaling of the IL-1 and RANK receptors also in the absence of infection [87]. 

Osteoclasts express IL-1 receptors and their resorption activity is enhanced through the action of IL-1α and IL-1 β in the presence of RANKL. IL-1, as well as IL-18 and TNFα, can indirectly stimulate osteoclast formation through upregulation of RANKL production from T cells [88]. 

IL-1 receptors expressed on osteoclasts activate the TRAF6 pathway, already initiated by RANKL-RANK signaling, resulting in the further activation of NF-kB and NFATc1 and induction of marked osteoclast specific genes [89]. 

The nod-like receptor protein-3 inflammasome signaling pathway contributes to IL-1α and IL-1β activation and bone resorption. After inflammatory stimuli, the accumulated IL-1β triggers a series of inflammatory reactions and acts as a strong stimulator of bone resorption by increasing the expression of collagenolytic enzymes and matrix metalloproteinases, thus contributing to extracellular matrix degradation and in turn leading to bone resorption and osteoporosis. IL-1 also upregulates RANKL thus stimulating osteoclastogenesis, but also regulates the production of osteoprotegerin, a soluble RANKL receptor that prevents it from binding to RANK. In addition, IL-1 increases PGE2 synthesis in fibroblasts, indirectly inducing the expression of RANKL as well. IL-1 has, therefore, a long-lasting effect on osteoclastogenesis, which leads to bone resorption [90]. 

Removal of IL-1 or IL-1 receptor 1 in mouse knockout models has important effects on the bone microenvironment, resulting in reduced bone resorption, increased trabecular thickness, and skeletal growth, suggesting that silencing of central IL-1R signaling leads to progressive accrual of bone mass. Similarly, in animal models of rheumatoid arthritis, blocking IL-1 with either IL-1 receptor antagonists or anti-IL-1 monoclonal antibodies, protects bone and cartilage from progressive erosion and destruction. Moreover, clinical trials with the IL-1 receptor antagonist anakinra demonstrate that blocking the effects of IL-1 protects bone and cartilage in rheumatoid arthritis [91]. 

IL-1 signals downstream of the IL-1R through the adapter protein MyD88, which also transduces signals from various toll-like receptors (TLRs) after ligation by conserved microbial motifs known as pathogen-associated molecular patterns (PAMPs) [86]. 

Interestingly, osteoblasts and osteoclasts express innate immune receptors through which these cells sense and respond to PAMPs and inflammatory cytokines in cell culture [92]. Therefore, although MyD88 and IL-1R-dependent signaling pathways are necessary to control bacterial proliferation during osteomyelitis, these same pathways might also contribute to pathogen-induced bone loss through actions on skeletal cells. IL-1 dependent immune responses thus trigger collateral bone damage through activation of osteoclast mediated bone resorption.

ADP-ribosyltransferase diphtheria toxin-like 1 (ARTD1, formerly PARP1) regulates NF-κ B-induced IL-1β expression in osteoclasts, leading to autocrine regulation of the expression of osteoclast markers, such as NFATc1/A, by enzymatically altering the epigenetic status of the IL-1β promoter during sustained osteoclastogenesis [81].

IL-1 is also considered a potential prognostic biomarker for predicting breast cancer patients at increased risk for developing bone metastasis. In mouse models, IL-1 and its receptor are upregulated in bone metastatic breast cancer cells. In particular, IL-1 influences tumor growth and metastasis, either through direct proliferative effects or by promoting inflammatory and angiogenic pathways in surrounding host cells, as well as by facilitating bone resorption and skeletal invasion [93]. 

### 3.4. IL-33

Evidence has shown immunological control of bone remodeling. In addition to RAGE ligand alarmins, cytokines are also involved in the pathogenesis of osteoporosis. Bone cell activity is modulated by cytokines, but not all cytokines involved have an osteoclastogenic effect [2].

Among the interleukins involved in osteoporosis, interleukin 33 (IL-33) is considered an alarmin. 

IL-33 belongs to the IL-1 cytokine family, in which IL-1 and IL-18 have well-known actions on bone cells [94]. IL-33 is expressed by stromal cells, and its upregulation follows pro-inflammatory stimuli. It has different functions: as a traditional cytokine, as a nuclear factor regulating gene transcription, and as an alarmin. IL-33 interacts with the receptors ST2 (IL-1RL1) and IL-1 receptor accessory protein (IL-1RAcP), especially expressed by innate immune and T helper 2 (Th2) cells. Il-33 receptors have also been identified on Th1 lymphocytes, natural killer cells (NK), and regulatory T cells (Treg) [94]. Evidence shows a role of IL-33 in the pathogenesis of Th2-related diseases and in the regulation of adaptive and innate response [95,96,97].

IL-33 induces protective effects in inflammatory diseases. The protective or exacerbating aspects of the presence of IL-33 or its inhibitors depend on genetic predisposition, duration of disease (acute or chronic), dose or kinetics of IL-33, and the cytokine microenvironment. IL-33 acts at different levels (molecular, cellular, and transcriptional), mediating pluripotent functions in several diseases and, thus, having a potential therapeutic role in attenuating pathological processes [98].

The role of IL-33 in osteoporosis is debated. Some studies have shown contrasting roles of IL-33 in bone remodeling. As reported by some authors, IL-33 stops osteoclast formation from bone marrow precursor cells [99], while others showed IL-33 shifting the equilibrium in vitro from osteoclasts to the differentiation of alternatively activated macrophages [100]. A reduction in osteoprotegerin expression mediated by osteoblasts and an increase in osteoclastogenic factor release inducing bone resorption during inflammation mediated by IL-33 was proposed [101], even though some in vitro evidence suggests that IL-33 stimulates matrix mineralization [102]. IL-33 stimulates osteoblast maturation and decreases osteoclastogenesis [103] by participating in Th2-mediated processes [104]. Promotion of osteoclast differentiation from human monocyte precursors regulated by lL-33, thus inducing bone resorption independent of the RANKL pathway, has been proposed [105]. Moreover, IL-33 inhibition of RANKL-dependent osteoclast formation [106] has been shown. Similarly, other evidence shows a suppressive effect of IL-33 on osteoclast differentiation via the inactivation of a key regulator of RANKL-induced osteoclast formation, the nuclear factor of activated T-cell cytoplasmic 1 [107]. Moreover, experimental studies suggest that mice missing the IL-33 receptor show a low bone mass and an increase in osteoclast formation [108]. 

We also previously demonstrated in postmenopausal women, the anti-osteoclastic effect of IL-33, but the direct effect of IL-33 on osteoclast function or bone resorption remain unclear. Our results showed a negative correlation between IL-33 and the resorption marker carboxyterminal telopeptide of collagen I, confirming an inhibition of osteoclast differentiation mediated by IL-33 [10].

It has been proposed that IL-33 also inhibits osteoclast differentiation by inducing anti-osteoclastogenic cytokines such as IL-4, IL-10, IFN-y, and granulocyte-macrophage colony-stimulating factor, thus leading to the differentiation of osteoclast precursors toward dendritic cells and alternatively activated macrophages [100].

Finally, a unique role of the IL-1 family of cytokines in modulating the reprogramming potential of Treg cells during inflammation has been recently identified. In particular, IL-1 and IL-33 are two distinct members of the IL-1 family of cytokines which play opposing roles in dictating the dynamics and functional specialization of Treg cells during immune challenges and chronic inflammation. Cell surface expression of the IL-33 receptor ST2 identifies a subset of functionally stable Foxp3+ Treg cells that are resistant to plasticity and loss of suppressive function, whereas IL-1 receptor I (IL-1RI) expression identifies Treg cells that acquire a Th17 cell phenotype and fail to suppress. Therefore, the production of IL-1β by dendritic cells promotes Th17 differentiation which contributes to the development of osteoporosis [109].

## 4. Conclusions

**Figure 1:**
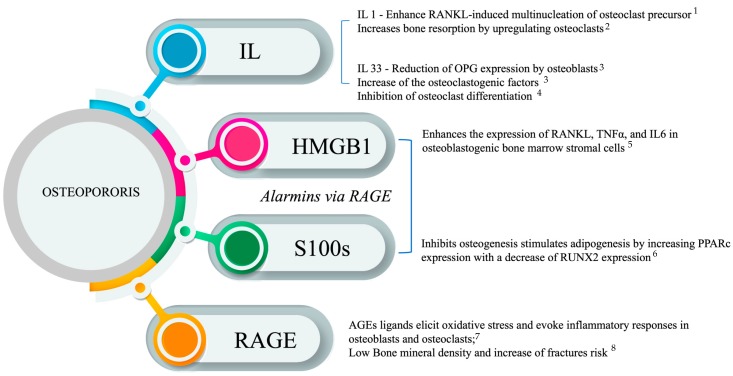
Proposed mechanism of alarmins. (1) Involvement in osteoporosis. Robaszkiewicz et al. 2016, [81]; (2) De Martinis et al., 2020, [84]; (3) Schulze et al., 2011 [101]; (4) Saleh et al., 2011 [103]; (5) Feng et al., 2016, [32]; (6) Li et al., 2016, [34]; (7) Zhou and Xiong, 2011 [24]; (8) Yamamoto et al., 2009. [21].

Alarmins have important immune functions in normal and pathological conditions. During chronic damage and metabolic disturbances, these proteins are, in fact, an integral part of complex proinflammatory events, sharing regulatory mechanisms leading to tissue repair or damage and organ dysfunction. RAGE and the IL-33, HMBG1, and S100 alarmins have important physiological functions in skeletal remodeling. The therapeutic potential of these endogenous molecules or their antagonists could constitute a pharmacological target aiming to prevent bone loss and skeletal fragility induced by aging and inflammation. According to the proposed mechanism by which RAGE and alarmins are involved in osteoporosis (Figure 1), most of the current research in this field is therefore primarily aimed at the construction of new potential inhibitor and/or agonist drugs capable of modulating alarmin signals. RAGE and its ligands HMGB and S100 alarmins, as well as IL-33, could also constitute potential biomarkers of bone disease progression and severity and could be useful in the diagnosis and follow-up of patients affected by osteoporosis. Therefore, although recent advances in osteoimmunology have conclusively demonstrated how alterations in the levels of RAGE and its ligands are clearly involved in the pathogenesis of osteoporosis, their specific roles and their reciprocal interactions in the pathogenesis of bone disease are not yet fully understood. However, their local and systemic roles are intricate, depending on a variety of complex factors such as the engagement of differential receptors, the type of cell involved, and their mutual interactions. Therefore, the targeting of these endogenous mediators in osteoporosis remains challenging.

## References

[1] Calleja-Agius J., Brincat M., Genazzani A.R., Brincat M. (2014). Menopause-Related Changes in the Musculoskeletal System, Cartilages and Joints. Frontiers in Gynecological Endocrinology.

[2] Ciccarelli F., De Martinis M., Ginaldi L. (2015). Glucocorticoids in patients with rheumatic diseases: Friends or enemies of bone?. Curr. Med. Chem..

[3] Ginaldi L., De Martinis M. (2016). Osteoimmunology and beyond. Curr. Med. Chem..

[4] Ginaldi L., De Martinis M., Ciccarelli F., Saitta S., Imbesi S., Mannucci C., Gangemi S. (2015). Increased levels of interleukin 31 (IL-31) in osteoporosis. BMC Immunol..

[5] Giovos G., Yavropoulou M.P., Yovos J.G. (2019). The role of cellular senescence in diabetes mellitus and osteoporosis: Molecular pathways and potential interventions. Hormones.

[6] De Martinis M., Sirufo M.M., Ginaldi L. (2019). Osteoporosis: Current and emerging therapies targeted to immunological checkpoints. Curr. Med. Chem..

[7] Rider P., Voronov E., Dinarello C.A., Apte R.N., Cohen I. (2017). Alarmins: Feel the Stress. J. Immunol..

[8] Bianchi M.E. (2007). DAMPs, PAMPs and alarmins: All we need to know about danger. J. Leukoc. Biol..

[9] Bertheloot D., Latz E. (2017). HMGB1, IL-1α, IL-33 and S100 proteins: Dual-function alarmins. Cell. Mol. Immunol..

[10] Kapurniotu A., Gokce O., Bernhagen J. (2019). The Multitasking Potential of Alarmins and Atypical Chemokines. Front. Med..

[11] Ginaldi L., De Martinis M., Saitta S., Sirufo M.M., Mannucci C., Casciaro M., Ciccarelli F., Gangemi S. (2019). Interleukin-33 serum levels in postmenopausal women with osteoporosis. Sci. Rep..

[12] Plotkin L.I., Essex A.L., Davis H.M. (2019). RAGE Signaling in Skeletal Biology. Curr. Osteop. Rep..

[13] Dattagupta A., Sathyamurthy I. (2018). ST2: Current status. Ind. Heart. J..

[14] Macari S., Madeira M.F.M., Lima I.L.A., Pereira T.S.F., Dias G.J., Cirelli J.A., de Molon R.S., Fukada S.Y., Szawka R.E., Garlet G.P. (2018). ST2 regulates bone loss in a site-dependent and estrogen-dependent manner. J. Cell. Biochem..

[15] Reynaert N.L., Gopal P., Rutten E.P.A., Wouters E.F.M., Schalkwijk C.G. (2016). Advanced glycation end products and their receptor in age-related, non-communicable chronic inflammatory diseases; Overview of clinical evidence and potential contributions to disease. Int. J. Biochem. Cell. Biol..

[16] Hanford L.E., Enghild J.J., Valnickova Z., Petersen S.V., Schaefer L.M., Schaefer T.M., Reinhart T.A., Oury T.D. (2004). Purification and characterization of mouse soluble nreceptor for advanced glycation end products (sRAGE). J. Biol. Chem..

[17] Hudson B.I., Lippman M.E. (2018). Targeting RAGE signaling in inflammatory disease. Annu. Rev. Med..

[18] Rojas A., Morales M., Gonzalez I., Araya P. (2019). Inhibition of RAGE axis signaling: A pharmacological challenge. Curr. Drug. Targets..

[19] Chuah Y.K., Basir R., Talib H., Tie T.H., Nordin N. (2013). Receptor for advanced glycation end products and its involvement in inflammatory diseases. Int. J. Inflam..

[20] Raska I., Raskova M., Zikan V., Skrha J. (2017). Prevalence and risk factors of osteoporosis in postmenopausal women with type 2 diabetes mellitus. Cent. Eur. J. Public Health.

[21] Yamamoto M., Yamaguchi T., Yamauchi M., Sugimoto T. (2009). Low serum level of the endogenous secretory receptor for advanced glycation end products (esRAGE) is a risk factor for prevalent vertebral fractures independent of bone mineral density in patients with type 2 diabetes. Diabetes Care.

[22] Galliera E., Marazzi M.G., Gazzaruso C., Gallotti P., Coppola A., Montalcini T., Pujia A., Corsi Romanelli M.M. (2017). Evaluation of circulating sRAGE in osteoporosis according to BMI, adipokines and fracture risk: A pilot observational study. Immun. Ageing.

[23] Braley A., Kwak T., Jules J., Harja E., Landgraf R., Hudson B.I. (2016). Regulation of receptor for advanced glycation end products (RAGE). Ectodomain shedding and its role in cell function. J. Biol. Chem..

[24] Zhou Z., Xiong W.C. (2011). RAGE and its ligands in bone metabolism. Front. Biosci..

[25] Sorci G., Riuzzi F., Giambanco I., Donato R. (2013). RAGE in tissue homeostasis, repair and regeneration. Biochim. Biophys. Acta..

[26] Galliera E., Marazzi M.G., Vianello E., Drago L., Luzzati A., Bendinelli P., Maroni P., Tacchini L., Desiderio M.A., Corsi Romanelli M.M. (2016). Circulating sRAGE in the diagnosis of osteolytic bone metastasis. J. Biol. Regul. Homeost. Agents.

[27] Ramasamy R., Shekhtman A., Schmidt A.M. (2016). The multiple faces of RAGE—opportunities for therapeutic intervention in aging and chronic disease. Expert Opin. Ther. Targets.

[28] Yang H., Lundback P., Ottosson L., Erlandsson-Harris H., Venereau E., Bianchi M.E., Al-Abed Y., Andersson U., Tracey K.J., Antoine D.J. (2012). Redox modification of cysteine residues regulates the cytokine activity of high mobility group box-1 (HMGB1). Mol. Med..

[29] Bongarzone S., Savickas V., Luzi F., Gee A.D. (2017). Targeting the receptor for advanced glycation endproducts (RAGE): A medicinal chemistry perspective. J. Med. Chem..

[30] Wolf D., Anto-Michel N., Blankenbach H., Wiedemann A., Buscher K., Hohmann J.D., Lim B., Bäum M., Marki A., Mauler M. (2018). A ligand-specific blockade of the integrin Mac-1 selectively targets pathologic inflammation while maintaining protective host-defense. Nat. Commun..

[31] Meng H.Z., Zhang W.L., Liu F., Yang M.W. (2015). Advanced glycation end products affect osteoblast proliferation and function by modulating autophagy via the receptor of advanced glycation end products/Raf protein/mitogen-activated protein kinase/extracellular signal-regulated kinase kinase/extracellular signal-regulated kinase (RAGE/Raf/MEK/ERK) pathway. J. Biol. Chem..

[32] Feng L., Xue D., Chen E., Zhang W., Gao X., Yu J., Feng Y., Pan Z. (2016). HMGB1 promotes the secretion of multiple cytokines and potentiates the osteogenic differentiation of mesenchymal stem cells through the Ras/MAPK signaling pathway. Exp. Ther. Med..

[33] Asadipooya K., Uy E.M. (2019). Advanced glycation end products (AGEs), receptor for AGEs, diabetes, and bone: Review of the literature. J. Endocr. Soc..

[34] Li Z., Li C., Zhou Y., Chen W., Luo G., Zhang Z., Xu D., Sheng P. (2016). Advanced glycation end products biphasically modulate bone resorption in osteoclast-like cells. Am. J. Physiol. Endocrinol. Metab..

[35] Schmidt A.M., Yan S.D., Yan S.F., Stern D.M. (2000). The biology of the receptor for advanced glycation end products and its ligands. Biochim. Biophys. Acta.

[36] Byun K., Yoo Y., Son M., Lee J., Jeong G.B., Park Y.M., Salekdeh G.H., Lee B. (2017). Advanced glycation end-products produced systemically and by macrophages: A common contributor to inflammation and degenerative diseases. Pharmacol. Ther..

[37] Yamamoto M., Sugimoto T. (2016). Advanced glycation end products, diabetes, and bone strength. Curr. Osteoporos. Rep..

[38] Poundarik A.A., Wu P.C., Evis Z., Sroga G.E., Ural A., Rubin M., Vashishth D. (2015). A direct role of collagen glycation in bone fracture. J. Mech. Behav. Biomed. Mater..

[39] Lalla E., Lamster I.B., Feit M., Huang L., Spessot A., Qu W., Kislinger T., Lu Y., Stern D.M., Schmidt A.M. (2000). Blockade of RAGE suppresses periodontitis-associated bone loss in diabetic mice. J. Clin. Investig..

[40] Panza F., Seripa D., Solfrizzi V., Imbimbo B.P., Lozupone M., Leo A., Sardone R., Gagliardi G., Lofano L., Creanza B.C. (2016). Emerging drugs to reduce abnormal beta-amyloid protein in Alzheimer’s disease patients. Expert Opin. Emerg. Drugs.

[41] Deane R., Singh I., Sagare A.P., Bell R.D., Ross N.T., LaRue B., Love R., Perry S., Paquette N., Deane R.J. (2012). A multimodal RAGE-specific inhibitor reduces amyloid betamediated brain disorder in a mouse model of Alzheimer disease. J. Clin. Investig..

[42] Zhang M., Li Y., Rao P., Huang K., Luo D., Cai X., Xiao J. (2018). Blockade of receptors of advanced glycation end products ameliorates diabetic osteogenesis of adipose-derived stem cells through DNA methylation and Wnt signalling pathway. Cell Prolif..

[43] Cheng Y.Z., Yang S.L., Wang J.Y., Ye M., Zhuo X.Y., Wang L.T., Chen H., Zhang H., Yang L. (2018). Irbesartan attenuates advanced glycation end products-mediated damage in diabetes-associated osteoporosis through the AGEs/RAGE pathway. Life Sci..

[44] Yamagishi S.I., Matsui T. (2018). Therapeutic Potential of DNA-aptamers Raised Against AGE-RAGE Axis in Diabetes-related Complications. Curr. Pharm. Des..

[45] Willett T.L., Pasquale J., Grynpas M.D. (2014). Collagen modifications in postmenopausal osteoporosis: Advanced glycation endproducts may affect bone volume, structure and quality. Curr. Osteoporos. Rep..

[46] Yang D., Han Z., Oppenheim J.J. (2017). Alarmins and Immunity. Immumol. Rev..

[47] Bonaldi T., Langst G., Strohner R., Becker P.B., Bianchi M.E. (2002). The DNA chaperone HMGB1 facilitates ACE/CHRAC-dependent nucleosome sliding. EMBO J..

[48] Muller S., Ronfani L., Bianchi M.E. (2004). Regulated expression and subcellular localization of HMGB1, a chromatin protein with a cytokine function. J. Intern. Med..

[49] Singh J., Dixon G.H. (1990). High mobility group proteins 1 and 2 function as general class II transcription. Am. Chem. Soc..

[50] Bonaldi T., Talamo F., Scaffidi P., Ferrera D., Porto A., Bachi A., Rubartelli A., Agresti A., Bianchi M.E. (2003). Monocytic cells hyperacetylate chromatin protein HMGB1 to redirect it towards secretion. EMBO J..

[51] Scaffidi P., Misteli T., Bianchi M.E. (2002). Release of chromatin protein HMGB1 by necrotic cells triggers inflammation. Nature.

[52] Lee D., Taniguchi N., Sato K., Choijookhuu N., Hishikawa Y., Kataoka H., Morinaga H., Lotz M., Chosa E. (2018). HMGB2 is a novel adipogenic factor that regulates ectopic fat infiltration in skeletal muscles. Sci. Rep..

[53] Ronfani L., Ferraguti M., Croci L., Ovitt C.E., Scholer H.R., Consalez G.G., Bianchi M.E. (2001). Reduced fertility and spermatogenesis defects in mice lacking chromosomal protein HMGB2. Development.

[54] Agresti A., Bianchi M.E. (2003). HMGB proteins and gene expression. Curr. Opin. Genet. Dev..

[55] De Martinis M., Franceschi C., Monti D., Ginaldi L. (2007). Apoptosis remodeling in immunosenescence: Implications for strategies to delay ageing. Curr. Med. Chem..

[56] Taniguchi N., Yoshida K., Ito T., Tsuda M., Mishima Y., Furumatsu T., Ronfani L., Abeyama K., Kawahara K., Komiya S. (2007). Stage-specific secretion of HMGB1 in cartilage regulates endochondral ossification. Mol. Cell. Biol..

[57] Li Q., Yu B., Yang P. (2015). Hypoxia-induced HMGB1 in would tissues promotes the osteoblast cell proliferation via activating ERK/JNK signaling. Int. J. Clin. Exp. Med..

[58] Hou C., Luan L., Ren C. (2018). Oxidized low-density lipoprotein promotes osteoclast differentiation from CD68 positive mononuclear cells by regulating HMGB1 release. Biochem. Biophys. Res. Commun..

[59] Franke S., Ruster C., Pester J., Hofmann G., Oelzner P., Wolf G. (2011). Advanced glycation end products affect growth and function of osteoblasts. Clin. Exp. Rheumatol..

[60] Yang J., Shah R., Robling A.G., Templeton E., Yang H., Tracey K.J., Bidwell J.P. (2008). HMGB1 is a bone-active cytokine. J. Cell. Physiol..

[61] Chen M.Q., Luan J.J. (2019). HMGB1 promotes bone fracture healing through activation of ERK signaling pathway in a rat tibial fracture model. Kaohsiung J. Med. Sci..

[62] Yamoah K., Brebene A., Baliram R., Inagaki K., Dolios G., Arabi A., Majeed R., Amano H., Wang R., Yanagisawa R. (2008). High-mobility group box proteins modulate tumor necrosis factor-alpha expression in osteoclastogenesis via a novel deoxyribonucleic acid sequence. Mol. Endocrinol..

[63] Zhou Z., Han J.Y., Xi C.X., Xie J.X., Feng X., Wang C.Y., Mei L., Xiong W.C. (2008). HMGB1 regulates RANKL-induced osteoclastogenesis in a manner dependent on RAGE. J. Bone Miner. Res..

[64] Wang H., Zhu S., Zhou R., Li W., Sama A.E. (2008). Therapeutic potential of HMGB1-targeting agents in sepsis. Exp. Rev. Mol. Med..

[65] Donato R., Cannon B.R., Sorci G., Riuzzi F., Hsu K., Weber D.J., Geczy C.L. (2013). Functions of S100 Proteins. Curr. Mol. Med..

[66] Kim H., Lee Y.D., Kim M.K., Kwon J.O., Song M.K., Lee Z.H., Kim H.H. (2017). Extracellular S100A4 negatively regulates osteoblast function by activating the NF-kappaB pathway. BMB Rep..

[67] Marshak D.R., Pena L.A. (1992). Potential role of S100 beta in Alzheimer’s disease: An hypothesis involving mitotic protein kinases. Prog. Clin. Biol. Res..

[68] Griffin W.S. (2006). Inflammation and neurodegenerative diseases. Am. J. Clin. Nutr..

[69] Foell D., Roth J. (2004). Proinflammatory S100 proteins in arthritis and autoimmune disease. Arthritis Rheum..

[70] Chano T., Ishizawa M., Matsumoto K., Morimoto S., Hukuda S., Okabe H. (1995). The identity of proliferating cells in bone tumors with cartilaginous components: Evaluation by double-immunohistochemical staining using proliferating cell nuclear antigen and S-100 protein. Eur. J. Histochem..

[71] Yammani R.R. (2012). S100 proteins in cartilage: Role in arthritis. Biochim. Biophys. Acta..

[72] Marenholz I., Heizmann C.W., Fritz G. (2004). S100 proteins in mouse and man: From evolution to function and pathology (including an update of the nomenclature). Biochem. Biophys. Res. Commun..

[73] Ostendorp T., Leclerc E., Galichet A., Koch M., Demling N., Weigle B., Heizmann C.W., Kroneck P.M., Fritz G. (2007). Structural and functional insights into RAGE activation by multimeric S100B. EMBO J..

[74] Hofmann M.A., Drury S., Fu C., Qu W., Taguchi A., Lu Y., Avila C., Kambham N., Bierhaus A., Nawroth P. (1999). RAGE mediates a novel proinflammatory axis: A central cell surface receptor for S100/calgranulin polypeptides. Cell.

[75] Duarte W.R., Shibata T., Takenaga K., Takahashi E., Kubota K., Ohya K., Ishikawa I., Yamauchi M., Kasugai S. (2003). S100A4: A novel negative regulator of mineralization and osteoblast differentiation. J. Bone Miner. Res..

[76] Grevers L.C., de Vries T.J., Vogl T., Abdollahi-Roodsaz S., Sloetjes A.W., Leenen P.J., Roth J., Everts V., van den Berg W.B., van Lent P.L. (2011). S100A8 enhances osteoclastic bone resorption in vitro through activation of Toll-like receptor 4: Implications for bone destruction in murine antigen-induced arthritis. Arthritis Rheum..

[77] Yoshida T., Flegler A., Kozlov A., Stern P.H. (2009). Direct inhibitory and indirect stimulatory effects of RAGE ligand S100 on sRANKLinduced osteoclastogenesis. J. Cell. Biochem..

[78] Li D., Zhang R., Zhu W., Xue Y., Zhang Y., Huang Q., Liu M., Liu Y. (2013). S100A16 inhibits osteogenesis but stimulates adipogenesis. Mol. Biol. Rep..

[79] Satoh T., Otsuka A., Contassot E., French L.E. (2015). The inflammasome and IL-1β: Implications for the treatment of inflammatory diseases. Immunotherapy.

[80] Cohen S.B. (2004). The use of anakinra, an interleukin-1 receptor antagonist, in the treatment of rheumatoid arthritis. Rheum. Dis. Clin. N. Am..

[81] Robaszkiewicz A., Qu C., Wisnik E., Ploszaj T., Mirsaidi A., Kunze F.A., Richards P.J., Cinelli P., Mbalaviele G., Hottiger M.O. (2016). ARTD1 regulates osteoclastogenesis and bone homeostasis by dampening NF-κBdependent transcription of IL-1β. Sci. Rep..

[82] Ohori F., Kitaura H., Ogawa S., Shen W.R., Qi J., Noguchi T., Marahleh A., Nara Y., Pramusita A.I. (2020). IL-33 Inhibits TNF-α-Induced Osteoclastogenesis and Bone Resorption. Int. J. Mol. Sci..

[83] Strand V., Kavanaugh A.F. (2004). The role of interleukin-1 in bone resorption in rheumatoid arthritis. Rheumatology.

[84] De Martinis M., Sirufo M.M., Suppa M., Ginaldi L. (2020). IL-33/IL-31 Axis in Osteoporosis. Int. J. Mol. Sci..

[85] Shiratori T., Kyumoto-Nakamura Y., Kukita A., Uehara N., Zhang J., Koda K., Kamiya M., Badawy T., Tomoda R., Xu X. (2018). IL-1b Induces Pathologically Activated Osteoclasts Bearing Extremely High Levels of Resorbing Activity: A Possible Pathological Subpopulation of Osteoclasts, Accompanied by Suppressed Expression of Kindlin-3 and Talin-1. J. Immunol..

[86] Putnam N.E., Fulbright L.E., Curry J.M., Ford C.A., Petronglo J.R., Hendrix A.S., Cassat J.E. (2019). MyD88 and IL-1R signaling drive antibacterial immunity and osteoclast-driven bone loss during Staphylococcus aureus osteomyelitis. PLoS. Pathog..

[87] Kim J.H., Jin H.M., Kim K., Song I., Youn B.U., Matsuo K., Kim N. (2009). The mechanism of osteoclast differentiation induced by IL-1. J. Immunol..

[88] Zwerina J. (2004). Single and combined inhibition of tumor necrosis factor, interleukin-1, and RANKL pathways in tumor necrosis factor–induced arthritis: Effects on synovial inflammation, bone erosion, and cartilage destruction. Arthritis Rheum..

[89] Dai S.M., Nishioka K., Yudoh K. (2004). Interleukin (IL) 18 stimulates osteoclast formation through synovial T cells in rheumatoid arthritis: Comparison with IL1b and tumour necrosis factor. Ann. Rheum. Dis..

[90] Cheng R., Wu Z., Li M., Shao M., Hu T. (2020). Interleukin-1β is a potential therapeutic target for periodontitis: A narrative review. Int. J. Oral Sci..

[91] Abramson S.B., Amin A. (2002). Blocking the effects of IL-1 in rheumatoid arthritis protects bone and cartilage. Rheumatology.

[92] Matta B.M., Turnquist H.R. (2016). Expansion of regulatory T cells in vitro and in vivo by IL-33. Methods Mol. Biol..

[93] Holen I., Lefley D.V., Francis S.E., Rennicks S., Bradbury S., Coleman R.E., Ottewell P. (2016). IL-1 drives breast cancer growth and bone metastasis in vivo. Oncotarget.

[94] Takayanagi H. (2015). Osteoimmunology in 2014: Two-faced immunology-from osteogenesis to bone resorption. Nat. Rev. Rheumatol..

[95] Villarreal D.O., Weiner D.B. (2014). Interleukin 33: A switch-hitting cytokine. Curr. Opin. Immunol..

[96] Lloyd C.M. (2010). IL-33 family members and asthma–bridging innate and adaptive immune responses. Curr. Opin. Immunol..

[97] Vaccaro M., Cicero F., Mannucci C., Calapai G., Spatari G., Barbuzza O., Cannavò S.P., Gangemi S. (2016). IL-33 circulating serum levels are increased in patients with non-segmental generalized vitiligo. Arch. Dermatol. Res..

[98] Vocca L., Di Sano C., Uasuf C.G., Sala A., Riccobono L., Gangemi S., Albano G.D., Bonanno A., Gagliardo R., Profita M. (2015). IL-33/ ST2 axis controls Th2/IL-31 and Th17 immune response in allergic airway diseases. Immunobiology.

[99] Arshad M.I., Khan H.A., Noel G., Piquet-Pellorce C., Samson M. (2016). Potential therapeutic aspects of alarmin cytokine interleukin 33 or its inhibitors in various diseases. Clin. Ther..

[100] Zaiss M.M., Kurowska-Stolarska M., Böhm C., Gary R., Scholtysek C., Stolarski B., Reilly J., Kerr S., Millar N.L., Kamradt T. (2011). IL-33 shifts the balance from osteoclast to alternatively activated macrophage differentiation and protects from TNF-α-mediated bone loss. J. Immunol..

[101] Schulze J., Bickert T., Beil F.T., Zaiss M.M., Albers J., Wintges KStreichert T., Klaetschke K., Keller J., Hissnauer T.N., Spiro A.S. (2011). Interleukin-33 is expressed in differentiated osteoblasts and blocks osteoclast formation from bone marrow precursor cells. J. Bone. Min. Res..

[102] da Luz F.A., Oliveira A.P., Borges D., Brígido P.C., Silva M.J. (2014). The physiopathological role of IL-33: New highlights in bone biology and a proposed role in periodontal disease. Med. Inflamm..

[103] Saleh H., Eeles D., Hodge J.M., Nicholson G.C., Gu R., Pompolo S., Gillespie M.T., Quinn J.M. (2011). Interleukin-33, a target of parathyroid hormone and oncostatin m, increases osteoblastic matrix mineral deposition and inhibits osteoclast formation *in vitro*. Endocrinology.

[104] Keller J., Catala-Lehnen P., Wintges K., Schulze J., Bickert T., Ito W., Horst A.K., Amling M., Schinke T. (2012). Transgenic overexpression of interleukin-33 in osteoblasts results in decreased osteoclastogenesis. Biochem. Biophys. Res. Commun..

[105] Miller A.M. (2011). Role of IL-33 in inflammation and disease. J. Inflamm..

[106] Kiyomiya H., Ariyoshi W., Okinaga T., Kaneuji T., Mitsugi S., Sakurai T., Habu M., Yoshioka I., Tominaga K., Nishihara T. (2015). IL-33 inhibits RANKL-induced osteoclast formation through the regulation of Blimp-1 and IRF-8 expression. Biochem. Biophys. Res. Commun..

[107] Mun S.H., Ko N.Y., Kim H.S., Kim J.W., Kim D.K., Kim A.R., Lee S.H., Kim Y.G., Lee C.K., Lee S.H. (2010). Interleukin-33 stimulates formation of functional osteoclasts from human CD14+ monocytes. Cell. Mol. Life Sci..

[108] Zhu X., Zhao Y., Jiang Y., Qin T., Chen J., Chu X., Yi Q., Gao S., Wang S. (2017). Dectin-1 signaling inhibits osteoclastogenesis via IL-33-induced inhibition of NFATc1. Oncotarget.

[109] Alvarez F., Istomine R., Shourian M., Pavey N., Al-Aubodah T.A.F., Qureshi S., Fritz J.H., Piccirillo C.A. (2019). The alarmins IL-1 and IL-33 differentially regulate the functional specialisation of Foxp3+ regulatory T cells during mucosal inflammation. Mucosal Immunol..

